# Pre‐miR‐27a rs895819 polymorphism and risk of diffuse large B‐cell lymphoma

**DOI:** 10.1002/jcla.23088

**Published:** 2019-12-03

**Authors:** Weiyan Tang, Haonan Xu, Dawei Ma, Rong Ma, Jianqiu Wu, Xinnian Yu, Jifeng Feng, Qizhan Liu

**Affiliations:** ^1^ The Affiliated Cancer Hospital of Nanjing Medical University & Jiangsu Cancer Hospital & Jiangsu Institute of Cancer Research Nanjing China; ^2^ School of Basic Medicine and Clinical Pharmacy China Pharmaceutical University Nanjing China; ^3^ Jiangsu Key Lab of Cancer Biomarkers, Prevention and Treatment Jiangsu Collaborative Innovation Center for Cancer, Personalized Medicine School of Public Health Nanjing Medical University Nanjing China

**Keywords:** DLBCL, Pre‐miR‐27a, SNP, TGFBR1

## Abstract

**Background:**

Recently, several studies have investigated the relationship between Pre‐miR‐27a rs895819 polymorphism and risk of various cancers. However, the relationship between rs895819 and diffuse large B‐cell lymphoma (DLBCL) has not been well known.

**Methods:**

In this study, we conducted a case‐control study to explore the role of Pre‐miR‐27a rs895819 in risk of DLBCL. The PCR‐TaqMan and luciferase assays and in vitro experiments were used to evaluate polymorphism function.

**Results:**

As a result, we found subjects carrying with rs895819 AG/GG genotype had a significantly decreased risk when compared with those carrying the AA genotype. Further qPCR assay showed that the DLBCL patients carrying AG/GG genotypes showed a lower level of mature miR‐27a when compared with patients carrying AA genotype. Moreover, miR‐27a levels were upregulated in DLBCL tissues compared with normal lymphoid tissues. Further in vitro experiments showed that miR‐27a might function as an oncogene through target TGFBR1. In addition, TGFBR1 overexpression rescues effects of miR‐27a inhibitor on DLBCL cells phenotypes.

**Conclusions:**

In conclusion, these findings indicate that rs895819 A > G might reduce the expression of mature miR‐27a, and leading a higher level of TGFBR1, ultimately inhibiting the development of DLBCL.

## 1 INTRODUCTION

Diffuse large B‐cell lymphoma (DLBCL) is the most common subtype of non‐Hodgkin lymphoma (NHL), accounting for 30%‐40% of all the newly diagnosed cases.^1^ Mature B‐cell lymphoma is collectively the 10th most common cancer worldwide, with nearly 386,000 new diagnoses annually (2012 Cancer Research UK statistics), and of these 40% are DLBCL. In recent years, many studies have confirmed that genetic factors, especially the single nucleotide polymorphism (SNP), are closely associated with the susceptibility of DLBCL.^2^, ^3^, ^4^

MicroRNAs (miRNAs) are 17 to 25‐nucleotide noncoding RNAs that can modulate the expression of target genes. Accumulating evidence has shown that miRNA might play critical roles in cancer initiation and the progression processes,^5^ including DLBCL.^6^ MiR‐27a, which located at chromosome 19, has been reported to be associated with several types of cancer, such as colon cancer,^7^ breast cancer,^8^ gastric adenocarcinoma,^9^ and pancreatic carcinoma.^10^ In addition, several studies have investigated the relationship between Pre‐miR‐27a rs895819 polymorphism and risk of cancer such as colorectal cancer,^11^ cervical cancer,^12^ and gastric cancer.^13^ For example, Sun et al showed that rs895819 A > G might affect the expression of mature miR‐27a, leading to an increased level of ZBTB10, ultimately contributing to cancer risk.^14^ However, the relationship between rs895819 and DLBCL has not been well known.

In this study, we aimed to assess the association of the rs895819 polymorphism with risk of DLBCL in a Chinese population and uncover the potential mechanism.

## 2 MATERIALS AND METHODS

### 2.1 Patients

The institutional review boards of the Nanjing Medical University approved this study. All the enrolled subjects signed an informed consent before recruitment. Briefly, a total of 409 newly diagnosed and histologically confirmed DLBCL patients were consecutively recruited from Jiangsu province between 2011 and 2012. Cases were excluded if they had medical history of other cancers. The healthy controls (477) were randomly selected from individuals who were seeking for physical examinations in the same district and were frequency‐matched by age (±5 years) and sex. The controls were genetically unrelated to the cases.

### 2.2 Genotyping

The genomic DNA was isolated from the lymphocyte pellets from venous blood using the RelaxGene Blood DNA System (Tiangen biotech). The genotypes of the rs895819 polymorphism were detected using the TaqMan allelic discrimination assays equipped with ABI 7900HT Real‐Time Polymerase Chain Reaction (RT‐PCR) System (Applied Biosystems). The negative control was the loading well without DNA. We randomly selected a minimal 10% of the samples to repeat the results, and the accordance rate was 100%. The sequence for the rs895819 probe are as follows: FAM‐CAGGGTCCACGCCA‐MGB, HEX‐AGGGTCCACACCAA‐MGB.

### 2.3 qRT‐PCR

Total RNA from the tissue sample was extracted using Trizol RNA isolation reagent (Invitrogen) according to the manufacturer's protocol. The expression levels of miR‐27a were amplified with PCR primers (RiboBio) on the ABI 7900HT Real‐Time PCR System (Applied Biosystems). Real‐time PCR was performed with Power SYBR Green PCR Master Mix (Applied Biosystems Inc). All real‐time reactions were performed in triplicate.

### 2.4 Cell culture and transfection

OCI‐LY18 and OCI‐LY19 cells were cultured in RPMI‐1640 medium containing 10% fetal bovine serum (FBS) in 5% CO_2_ at 37°C. All media contained penicillin (100 U mL^−1^)/streptomycin (100 μg mL^−1^) (HyClone).

The miR‐27a mimics and miR‐27a inhibitors were synthesized by RiboBio. TGFBR1 overexpression vector containing or missing TGFBR1 3′UTR was constructed based on the pcDNA3.1. Transfections were carried out with the Lipofectamine 2000 reagent (Invitrogen). The cells were harvested 48h after transfection.

### 2.5 Analysis of cell proliferation, invasion, migration ability, and apoptosis

Cell proliferation was assessed by CCK8 assay, and the absorbance was measured at 450 nm. For the cell invasion assays, 2 × 10^4^ cells were plated into the Transwell insert coated with Matrigel (CytoSelect 24‐Well Cell Invasion Assay Kit; Cell Biolabs) and cultured with complete medium for 24 hours. For cell migration assays, 1 × 10^4^ cells were plated into the Transwell insert and cultured with complete medium for 24h. For detection of apoptosis, cells were stained with Annexin V‐FITC/PI at room temperature in the dark for 15 minutes. The rate of apoptosis was evaluated by Flow cytometry. All assays were independently performed in triplicates.

### 2.6 Western blot assay

Briefly, 20ug total protein was loaded in each lane and transferred to a PVDF membrane (Bio‐Rad). The membrane was then blocked with 5% non‐fat dried milk and incubated overnight at 4°C with TGFBR1(Abcam) and β‐actin (1:2000 dilution; CMCTAG) primary antibody and incubated with horseradish peroxidase (HRP)‐conjugated goat secondary antibodies for 1 hour at room temperature. Protein bands were finally visualized by enhanced chemiluminescence (ECL) and detected using the ECL Detection System (Amersham Pharmacia Biotech).

### 2.7 Dual‐luciferase reporter assay

Reporter plasmids were constructed based on the reporter vector psiCHECK‐2 (Promega). Briefly, reporter plasmids along with miR‐27a mimics or inhibitors were co‐transfected into DLBCL cells using the Lipofectamine 2000 reagent (Invitrogen). After 24 hours, Firefly and Renilla Luciferase activities were measured using Dual‐Luciferase Reporter Assay System (Promega).

### 2.8 Statistical analysis

Hardy‐Weinberg equilibrium (HWE) analysis was performed by comparing the observed and expected genotype frequencies using chi‐square test. We evaluated the differences between cases and controls in demographic characteristics and genotype results of rs895819 by chi‐square test. The association between rs895819 and risk of DLBCL was estimated using odds ratios (ORs) and 95% confidence intervals (CIs). A two‐sided *P*‐value < .05 was considered statistically significant. All statistical analyses were performed using SAS software (version 9.1.3, SAS Institute).

## 3 RESULTS

### 3.1 Demographic and clinical characteristics of the study subjects

The basic characteristics of cases and controls in our study are shown in Table 1. There were no differences in the distribution of age and gender between the cases and controls (age, *P* = .6228; gender, *P* = .8528; Table 1). The table showed that proportion of GCB and Non‐GCB of pathological type in cases was 33.5% and 66.5%, respectively. The rates of I + II and III + IV of Ann Arbor stage in cases were 45.2% and 54.8%, respectively. The rates of normal and evaluated of LDH in cases were 46.2% and 53.8%, respectively.

**Table 1:** Frequency distribution of the selected variables in DLBCL cases and controls

Variables	Case (N = 409)		Controls(N = 477)		*P*
	n	%	n	%	
Age					
≤60	268	65.5	305	63.9	.6228
>60	141	34.5	172	36.1	
Gender					
Male	241	58.9	284	59.5	.8528
Female	168	41.1	193	40.5	
Pathological type					
GCB	137	33.5			
Non‐GCB	272	66.5			
Ann Arbor stage					
I + II	185	45.2			
III + IV	224	54.8			
Performance status					
ECOG 0‐1	301	73.6			
ECOG 2‐4	108	26.4			
Extra nodal site					
<2	352	86.1			
≥2	57	13.9			
LDH					
Normal	189	46.2			
Elevated	220	53.8			
IPI status					
0‐2	301	73.6			
≥3	108	26.4			

### 3.2 Rs895819 polymorphism and DLBCL susceptibility

The genotype frequencies of rs895819 polymorphism in controls did not significantly deviate from Hardy‐Weinberg equilibrium model (*P* = .7005, Table 2). We found that the rs895819 genotypes and alleles distributions were significantly associated with the risk of DLBCL (*P* = .0044 and 0.0016, respectively; Table 2). When the AA genotype used as the reference, the heterozygous genotype AG was not associated with risk of DLBCL (adjusted OR = 0.76, 95% CI = 0.57‐1.00; Table 2), whereas the homozygous mutant GG genotype was associated with a significantly reduced risk of DLBCL (adjusted OR = 0.42, 95% CI = 0.24‐0.75; Table 2). In addition, the dominant genetic model was also associated with a reduced risk of DLBCL when combined AG and GG genotype (adjusted OR = 0.73, 95% CI = 0.53‐0.91; Table 2).

**Table 2:** Association between rs895819 polymorphisms and DLBCL risk

Genotype	Cases		Controls		*P*	Adjusted OR (95% CI)^a^
	n	%	n	%		
AA	234	57.2	230	48.2	.0044	
AG	157	38.4	205	43		0.76 (0.57‐1.00)
GG	18	4.4	42	8.8		0.42 (0.24‐0.75)
AG/GG	175	42.8	247	51.8	.0075	0.70 (0.53‐0.91)
G allele	0.2359			0.3029	.00158	
HWE	0.1909			0.7005		
*P* trend					.00132	

a Adjusted for age, gender in the logistic regression model.

### 3.3 Stratified analysis of rs895819 polymorphism and DLBCL risk

The association between the rs895819 polymorphism and the risk of DLBCL was further evaluated by stratification analysis. The results showed the risk of DLBCL was significantly associated with in the subgroups of age ≤60, normal LDH, III + IV Ann Arbor stage, and 0‐2 IPI (*P* = .0048, 0.0048, 0.0029, 0.0016, respectively, adjusted OR = 0.62, 0.61, 0.61, 0.65, respectively; 95% CI = 0.45‐0.87, 0.49‐0.88, 0.44‐0.84, 0.44‐0.87, respectively; Table 3). A similar result was observed in the subgroups of Non‐GCB pathological type, ECOG 0‐1 performance status and extranodal site <2 (*P* = .0093, 0.0091, respectively; adjusted OR = 0.67, 0.69, respectively, 95% CI = 0.50‐0.91, 0.51‐0.92, respectively; Table 3).

**Table 3:** Stratified analysis of rs895819 genotypes associated with DLBCL risk

Variables	Genotypes (cases/controls)		*P*	Adjusted OR (95% CI)^a^
	AA	AG/GG		
Age				
≤60	165/152	103/153	.0048	0.62 (0.45‐0.87)
>60	69/78	72/94	.5269	0.87 (0.56‐1.36)
Gender				
Male	140/135	101/149	.0158	0.66 (0.47‐0.94)
Female	94/95	74/98	.2016	0.76 (0.50‐1.15)
Pathological type				
GCB	76/230	61/247	.1343	0.76 (0.52‐1.11)
Non‐GCB	158/230	114/247	.0093	0.67 (0.50‐0.91)
Ann Arbor stage				
I + II	99/230	86/247	.2214	0.82 (0.58‐1.15)
III + IV	135/230	89/247	.0029	0.61 (0.44‐0.84)
Performance status				
ECOG 0‐1	174/230	127/247	.0091	0.69 (0.51‐0.92)
ECOG 2‐4	60/230	48/247	.1685	0.73 (0.48‐1.12)
Extra nodal site				
<2	202/230	150/247	.009	0.70 (0.53‐0.92)
≥2	32/230	25/247	.2581	0.72 (0.41‐1.25)
LDH				
Normal	114/230	75/247	.0048	0.61 (0.44‐0.87)
Elevated	120/230	100/247	.3243	0.78 (0.57‐1.08)
IPI status				
0‐2	180/230	121/247	.0016	0.65 (0.49‐0.88)
≥3	54/230	54/247	.7379	0.87 (0.56‐1.34)

a Adjusted for age, gender in the logistic regression model.

### 3.4 Rs895829 polymorphism and miR‐27a expression

**Figure 1:**
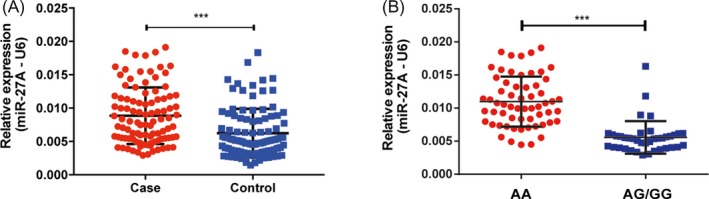
Expression of miR‐27a in DLBCL tissues. A, Expression of miR‐27a in DLBCL tissues and normal tissues. (n = 100 each, ***P < .001, compared to control group, two‐sided *t* tests, Error bars, SD) B, Relationship between re895819 genotypes and expression of miR‐27a. (n = 60, 35, and 5, for AA, AG, and GG, respectively, ***P < .001, compared to AA genotype, two‐sided *t* tests, Error bars, SD)

We detected miR‐27a levels in DLBCL tissues and paired normal tissues. Results showed that miR‐27a levels were upregulated in DLBCL tissues compared with normal tissues (Figure 1A). We then evaluated the association between rs895819 genotypes and the expression levels of miR‐27a. Of the 100 recruited DLBCL patients, the frequency distribution of rs895819 AA, AG, and GG was 60, 35, and 5, respectively. As shown in Figure 1B, we found the DLBCL patients carrying AG/GG genotypes showed a lower level of mature miR‐27a when compared with patients carrying AA genotype.

### 3.5 Effects of miR‐27a on DLBCL cell phenotypes

**Figure 2:**
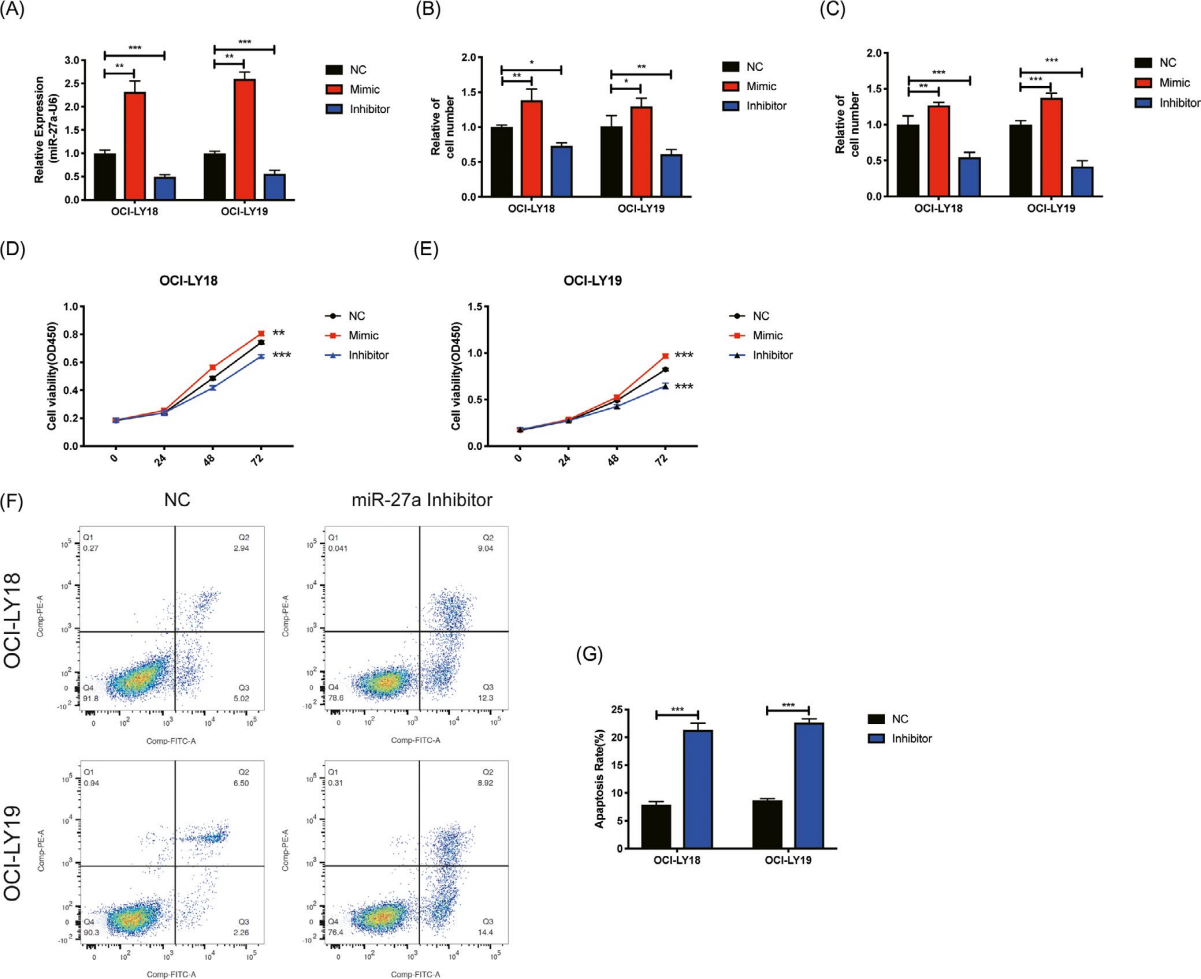
Effects of miR‐27a on DLBCL cell phenotypes. A, The expression of miR‐27a after transfection with miR‐27a mimic or inhibitor in DLBCL cells. (n = 3 each, **P < .01, ***P < .001, compared to NC group, one‐way ANOVA, Error bars, SD). B,C, Cell migration (B) and invasion (C) assay of DLBCL cells transfection with miR‐27a mimic or inhibitor. (n = 3 each, ***P < .001, compared to NC group, one‐way ANOVA, Error bars, SD). D,E, Cell viability in OCI‐LY18 (D) and OCI‐LY19 (E) cells transfection with miR‐27a mimic or inhibitor. (n = 3 each, **P < .01, ***P < .001, compared to NC group, one‐way ANOVA, Error bars, SD). F,G, Cell apoptosis rate in OCI‐LY18 and OCI‐LY19 cells transfection with miR‐27a mimic or inhibitor. (n = 3 each, ***P < .001, compared to NC group, two‐sided *t* tests, Error bars, SD)

In order to seek the function of miR‐27a in DLBCL, we then transfected miR‐27a mimic and inhibitor into OCI‐LY18 and OCI‐LY19 cell. As shown in Figure 2A, expression level of miR‐27a in DLBCL cells was significantly increased and decreased when treated with miR‐27a‐3p mimic and inhibitor, respectively. Furthermore, DLBCL cells treated with miR‐27a‐3p inhibitor showed decreased migration (Figure 2B), invasion (Figure 2C), and proliferation ability (Figure 2D,E), as well as increased apoptosis rate (Figure 2F,G). DLBCL cells treated with miR‐27a‐3p mimic showed increased migration, invasion, and proliferation ability (Figure 2B‐E).

### 3.6 Effects of miR‐27a on TGFBR1 expression

**Figure 3:**
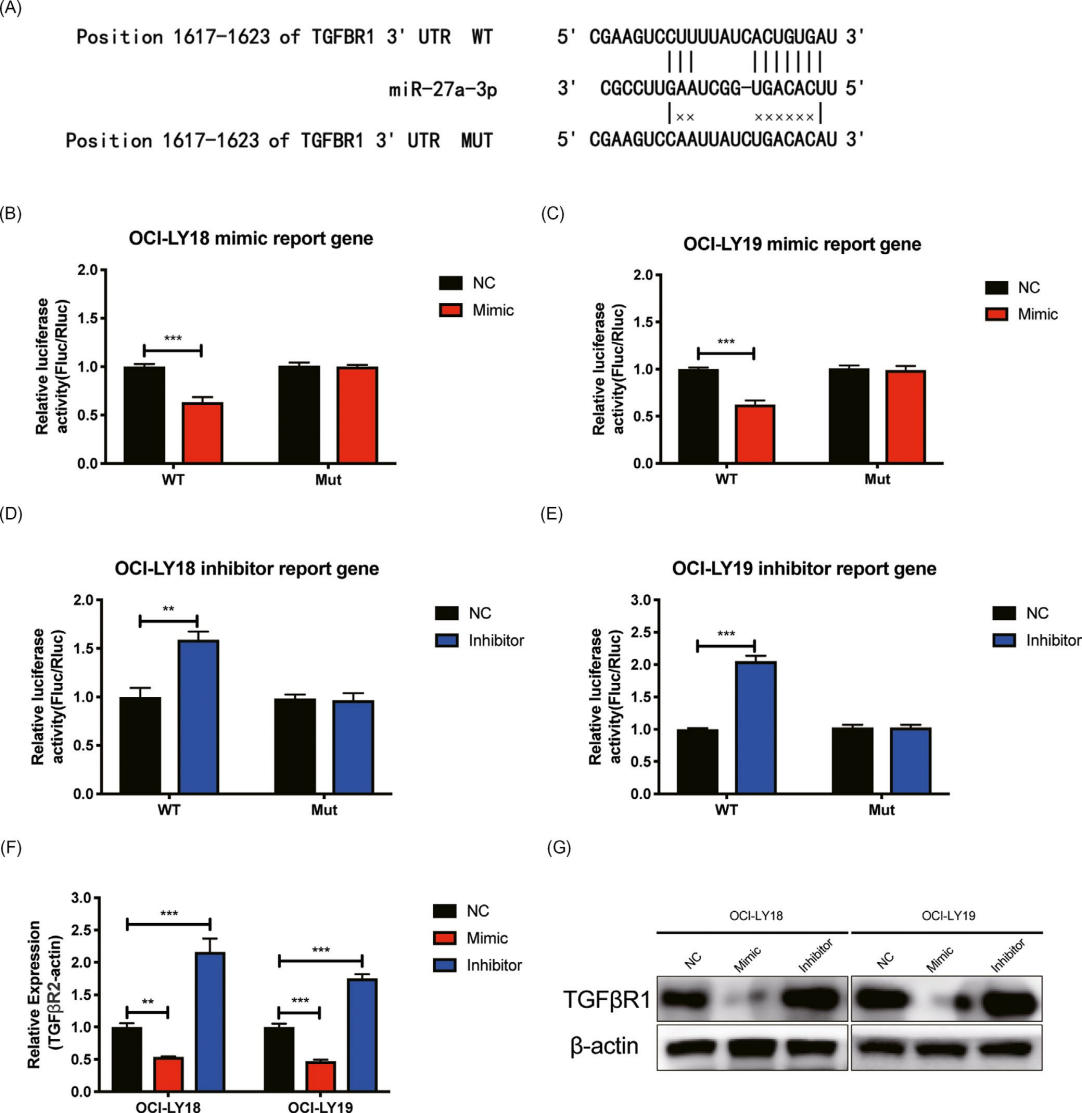
Effects of miR‐27a on TGFBR1 regulation. A, Predicted target sites of miR‐27a on TGFBR1 and the mutant sequence are shown. B,C, Relative reporter gene activity of psiCHECK‐2 containing mutant or wild type TGFBR1 3′UTR co‐transfected with miRNA control or miR‐27a mimics in OCI‐LY18 (B) and OCI‐LY19 (C) cells lines. (n = 3 each, ***P < .001, c compared to NC group, two‐sided *t* tests, Error bars, SD). D,E, Relative reporter gene activity of psiCHECK‐2 containing mutant or wild type TGFBR1 3′UTR co‐transfected with miRNA control or miR‐27a inhibitor in OCI‐LY18 (D) and OCI‐LY19 (E) cells lines. (n = 3 each, **P < .01, ***P < .001, c compared to NC group, two‐sided *t* tests, Error bars, SD). F,G, Expression of TGFBR1 mRNA (F) and Protein (G) in OCI‐LY18 and OCI‐LY19 lines co‐transfected with miRNA control, miR‐27a inhibitor or inhibitor. (n = 3 each, *** *P* < .001, compared to NC group, one‐way ANOVA, Error bars, SD)

To understand the functional mechanism by which miR‐27a modulates the malignant biological behavior of DLBCL cells, we then focused on identifying the direct targets of miR‐27a using Targetscan database (http://www.targetscan.org/vert_72/). We found that *TGFBR1* 3′UTR harbors a putative miR‐27a binding site. To verify whether *TGFBR1* is a bona fide target of miR‐27a, the wild and mutant type *TGFBR1* 3′UTR were then constructed in the psiCHECK‐2 vector (Figure 3A). The followed dual‐luciferase activity assay results showed luciferase activity in those two cells were significantly inhibited after co‐transfected with miR‐27a‐3p mimic and wild type of TGFBR1 3′UTR (Figure 3B,C) and increased after co‐transfected with miR‐27a‐3p inhibitor and wild type of TGFBR1 3′UTR (Figure 3D,E). In addition, results from RT‐qPCR and Western blot (Figure 3F,G) showed that both of TGFBR1 mRNA and protein levels were downregulated in cells treated with miR‐27a‐3p mimic and upregulated in cell treated with miR‐27a‐3p inhibitor.

### 3.7 TGFBR1 mediates regulation of miR‐27a on DLBCL cell phenotypes

**Figure 4:**
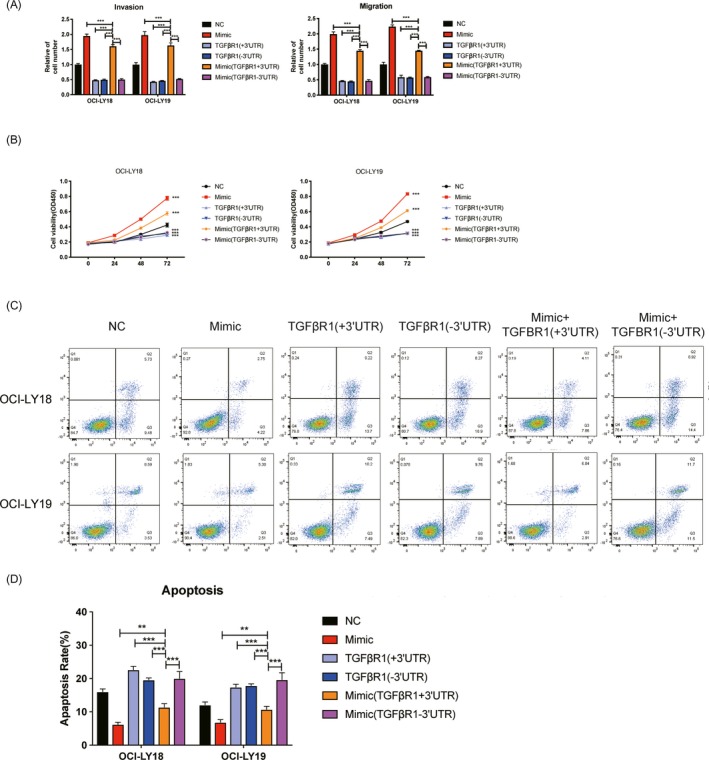
TGFBR1 mediates regulation of miR‐27a on DLBCL cell phenotypes. A, Cell migration(right) and invasion(left) assay of DLBCL cells transfection with miR‐27a mimic and TGFBR1 expression vector containing or missing 3′UTR. (n = 3 each, ***P < .001, compared to NC group, one‐way ANOVA, Error bars, SD). B, Cell viability of DLBCL cells transfection with miR‐27a mimic and TGFBR1 expression vector containing or missing 3′UTR. (n = 3 each, NC means no significance, ***P < .001, compared to NC group, one‐way ANOVA, Error bars, SD). C,D, Cell apoptosis rate of DLBCL cells transfection with miR‐27a mimic and TGFBR1 expression vector containing or missing 3′UTR. (n = 3 each, NC means no significance, ***P < .001, compared to NC group, one‐way ANOVA, Error bars, SD)

To further explore whether TGFBR1 mediates regulation of miR‐27a on DLBCL cell phenotypes, the TGFBR1 pcDNA3.1 expression vector contains or missing TGFBR1 3′UTR were constructed and co‐transfected with miR‐27a mimic, respectively. CCK‐8, flow cytometry, Transwell invasion, and migration assays were then used to examine the effects of TGFBR1 on DLBCL cells proliferation, apoptosis, and invasion through TGFBR1 restore. Compared with cells treated with miR‐27a mimic and expression vectors containing TGFBR1 3′UTR, cell migration, invasion (Figure 4A), and proliferation (Figure 4B) ability were significantly downregulated in both DLBCL cell transfected with TGFBR1 expression vectors missing 3′UTR only, TGFBR1 expression vectors containing TGFBR1 3′UTR only, and TGFBR1 expression vectors missing 3′UTR and miR‐27a mimic co‐transfected group. Cell apoptosis was significantly upregulated in both DLBCL cell transfected with TGFBR1 expression vectors missing 3′UTR only, TGFBR1 expression vectors containing TGFBR1 3′UTR only, and TGFBR1 expression vectors missing 3′UTR and miR‐27a mimic co‐transfected group (Figure 4C,D). In conclusion, those results indicated that TGFBR1 mediates regulation of miR‐27a on DLBCL cell phenotypes.

## 4 DISCUSSION

In our study, we found that subjects carrying with rs895819 AG/GG genotype had a significantly decreased risk when compared with those carrying the AA genotype. Further functional assay showed that rs895819 A > G might downregulate the expression of mature miR‐27a, and leading a high level of *TGFBR1*, ultimately inhibiting the development of DLBCL.

It has been reported that pre‐miR‐27a polymorphism was associated with various tumors, such as colorectal cancer,^15^, ^16^, ^17^ breast cancer,^18^, ^19^ cervical cancer,^12^ and renal cell cancer.^20^ However, to the best of our knowledge, no studies are focusing on the association between the SNPs in the pre‐miR‐27a and DLBDL risk. In our study, we found that rs895819 AG/GG genotypes had a significantly decreased the risk of DLBCL, and this association might be modulated by specific demographic factors. These results further suggest that DLBCL is a multistep and intricate process involving genetic and environment factors. We also observed a different association in the different clinic pathologic kinds of DLBCL, indicated that the cancer with different clinical feature might be regulated by different molecular mechanisms.

MiRNAs, one of novel posttranscriptional regulators, represent a small part of the genome, but they regulate nearly one‐third of human genes.^21^ Accumulating studies have shown that aberrant miRNA expressions are associated with various cancers.^22^ As an important member of miRNAs family, miR‐27a has been found to be involved in the development of multiple cancers by acting as an oncogene. Mertens‐Talcott et al showed that the oncogenic role of miR‐27a in breast cancer cells is due to the suppression of ZBTB10 and myt‐1.^8^ In gastric cancer, miR‐27a can inhibit cancer cell growth through regulating prohibitin levels.^9^ Yuan et al found that miR‐27a functions as an oncogene by targeting SPRY2 and regulates the growth, colony formation and migration of pancreatic cancer cells.^10^ In addition, previous study has shown miR‐27a was statistically upregulated in DLBCL compared to normal germinal cells.^23^ In our study, we observed that mir‐27a levels were upregulated in DLBCL tissues compared with normal tissues.

Previously study showed that TGF‐β signaling pathway controls different biological processes and dysregulation of its canonical members is associated with different cancer types.^24^ In malignant B lymphocytes, TGF‐β signaling had been reported as a tumor suppressor.^25^, ^26^ TGFBR1, one of the receptor ligand of TGF‐β, transduces the TGF‐beta signaling through phosphorylating SMAD2/3 or no canonical downstream components.^27^ In our study, we demonstrated that miR‐27a might directly target *TGFBR1* 3′UTR. In addition, *TGFBR1* overexpression rescues effects of miR‐27a inhibitor on DLBCL cells phenotypes. Taken together, these findings indicate the oncogenic role of miR‐27a/*TGFBR1* axis in development of DLBCL.

In conclusion, we have shown that rs895819 A > G could reduce the expression of mature miR‐27a, leading upregulating of TGFBR1, and ultimately inhibiting the development of DLBCL.

Undeniably, two limitations of this study should be addressed herein. First, only one stage case‐control study was performed in this study, in which the sample size might not be large enough to achieve sufficient statistical power. Second, only a CHB population‐based study was conducted in this study. Further studies with enough samples and different ethnic populations are required to confirm our findings.

## References

[1] Sehn LH , Gascoyne RD . Diffuse large B‐cell lymphoma: optimizing outcome in the context of clinical and biologic heterogeneity. Blood. 2015;125(1):22‐32. 25499448 10.1182/blood-2014-05-577189

[2] Zhuang H , Shen J , Zheng Z , Luo X , Gao R , Zhuang X . MicroRNA‐146a rs2910164 polymorphism and the risk of diffuse large B cell lymphoma in the Chinese Han population. Med Oncol. 2014;31(12):306. 25370733 10.1007/s12032-014-0306-z

[3] Essa ES , Alagizy HA . Association of MNS16A VNTR and hTERT rs2736098: G>A polymorphisms with susceptibility to diffuse large B‐cell lymphoma. Tumori. 2017;104(3):165‐171. 10.5301/tj.5000653 28967095

[4] Hu LL , Yu B , Yang J . MDR1 polymorphisms associated with risk and survival in diffuse large B‐cell lymphoma. Leuk Lymphoma. 2013;54(6):1188‐1193. 23088763 10.3109/10428194.2012.736980

[5] Wang J , Zhang KY , Liu SM , Sen S . Tumor‐associated circulating microRNAs as biomarkers of cancer. Molecules. 2014;19(2):1912‐1938. 24518808 10.3390/molecules19021912 PMC6271223

[6] Musilova K , Mraz M . MicroRNAs in B‐cell lymphomas: how a complex biology gets more complex. Leukemia. 2015;29(5):1004‐1017. 25541152 10.1038/leu.2014.351

[7] Bao Y , Chen Z , Guo Y , et al. Tumor suppressor microRNA‐27a in colorectal carcinogenesis and progression by targeting SGPP1 and Smad2. PLoS ONE. 2014;9(8):e105991. 25166914 10.1371/journal.pone.0105991 PMC4148394

[8] Mertens‐Talcott SU , Chintharlapalli S , Li X , Safe S . The oncogenic microRNA‐27a targets genes that regulate specificity protein transcription factors and the G2‐M checkpoint in MDA‐MB‐231 breast cancer cells. Can Res. 2007;67(22):11001‐11011. 10.1158/0008-5472.CAN-07-2416 18006846

[9] Liu T , Tang H , Lang Y , Liu M , Li X . MicroRNA‐27a functions as an oncogene in gastric adenocarcinoma by targeting prohibitin. Cancer Lett. 2009;273(2):233‐242. 18789835 10.1016/j.canlet.2008.08.003

[10] Ma Y , Yu S , Zhao W , Lu Z , Chen J . miR‐27a regulates the growth, colony formation and migration of pancreatic cancer cells by targeting Sprouty2. Cancer Lett. 2010;298(2):150‐158. 20638779 10.1016/j.canlet.2010.06.012

[11] Liu FF , Dear K , Huang L , et al. Association between microRNA‐27a rs895819 polymorphism and risk of colorectal cancer: a meta‐analysis. Cancer Genet. 2016;209(9):388‐394. 27751356 10.1016/j.cancergen.2016.08.003

[12] Xiong XD , Luo XP , Cheng J , Liu X , Li EM , Zeng LQ . A genetic variant in pre‐miR‐27a is associated with a reduced cervical cancer risk in southern Chinese women. Gynecol Oncol. 2014;132(2):450‐454. 24380734 10.1016/j.ygyno.2013.12.030

[13] Xu Q , Chen TJ , He CY , Sun LP , Liu JW , Yuan Y . MiR‐27a rs895819 is involved in increased atrophic gastritis risk, improved gastric cancer prognosis and negative interaction with Helicobacter pylori. Sci Rep. 2017;7:41307. 28150722 10.1038/srep41307 PMC5288699

[14] Sun Q , Gu H , Zeng Y , et al. Hsa‐mir‐27a genetic variant contributes to gastric cancer susceptibility through affecting miR‐27a and target gene expression. Cancer Sci. 2010;101(10):2241‐2247. 20666778 10.1111/j.1349-7006.2010.01667.x PMC11159034

[15] Jiang Y , Lin DH , Xu JP , Chen WX , Zheng SJ , Song L . Genotype GG of rs895819 functional polymorphism within miR‐27a might increase genetic susceptibility to colorectal cancer in Han Chinese population. J Clin Lab Anal. 2016;30(4):351‐355. 26302683 10.1002/jcla.21862 PMC6806710

[16] Bian Q , Chen JJ , Gu JP , Xu J . Association between pre‐miR‐27a functional polymorphism and risk of colorectal cancer in north Chinese Han population. OncoTargets Ther. 2015;8:3003‐3007. 10.2147/OTT.S89754 PMC4621200 26527885

[17] Wang Z , Sun X , Wang Y , Liu X , Xuan Y , Hu S . Association between miR‐27a genetic variants and susceptibility to colorectal cancer. Diagnostic Pathol. 2014;9:146. 10.1186/1746-1596-9-146 PMC4261532 25078482

[18] Morales S , Gulppi F , Gonzalez‐Hormazabal P , et al. Association of single nucleotide polymorphisms in Pre‐miR‐27a, Pre‐miR‐196a2, Pre‐miR‐423, miR‐608 and Pre‐miR‐618 with breast cancer susceptibility in a South American population. BMC Genet. 2016;17(1):109. 27421647 10.1186/s12863-016-0415-0 PMC4946190

[19] Zhang N , Huo Q , Wang X , et al. A genetic variant in pre‐miR‐27a is associated with a reduced breast cancer risk in younger Chinese population. Gene. 2013;529(1):125‐130. 23954879 10.1016/j.gene.2013.07.041

[20] Shi D , Li P , Ma L , et al. A genetic variant in pre‐miR‐27a is associated with a reduced renal cell cancer risk in a Chinese population. PLoS ONE. 2012;7(10):e46566. 23118855 10.1371/journal.pone.0046566 PMC3484143

[21] Carthew RW . Gene regulation by microRNAs. Curr Opin Genet Dev. 2006;16(2):203‐208. 16503132 10.1016/j.gde.2006.02.012

[22] Esquela‐Kerscher A , Slack FJ . Oncomirs ‐ microRNAs with a role in cancer. Nat Rev Cancer. 2006;6(4):259‐269. 16557279 10.1038/nrc1840

[23] Troppan K , Wenzl K , Pichler M , et al. miR‐199a and miR‐497 are associated with better overall survival due to increased chemosensitivity in diffuse large B‐cell lymphoma patients. Int J Mol Sci. 2015;16(8):18077‐18095. 26251897 10.3390/ijms160818077 PMC4581236

[24] Levy L , Hill CS . Alterations in components of the TGF‐beta superfamily signaling pathways in human cancer. Cytokine Growth Factor Rev. 2006;17(1–2):41‐58. 16310402 10.1016/j.cytogfr.2005.09.009

[25] Dong M , Blobe GC . Role of transforming growth factor‐beta in hematologic malignancies. Blood. 2006;107(12):4589‐4596. 16484590 10.1182/blood-2005-10-4169 PMC1895802

[26] Bakkebo M , Huse K , Hilden VI , Smeland EB , Oksvold MP . TGF‐beta‐induced growth inhibition in B‐cell lymphoma correlates with Smad1/5 signalling and constitutively active p38 MAPK. BMC Immunol. 2010;11:57. 21092277 10.1186/1471-2172-11-57 PMC3006362

[27] Vander Ark A , Cao J , Li X . TGF‐beta receptors: In and beyond TGF‐beta signaling. Cell Signal. 2018;52:112‐120. 30184463 10.1016/j.cellsig.2018.09.002

