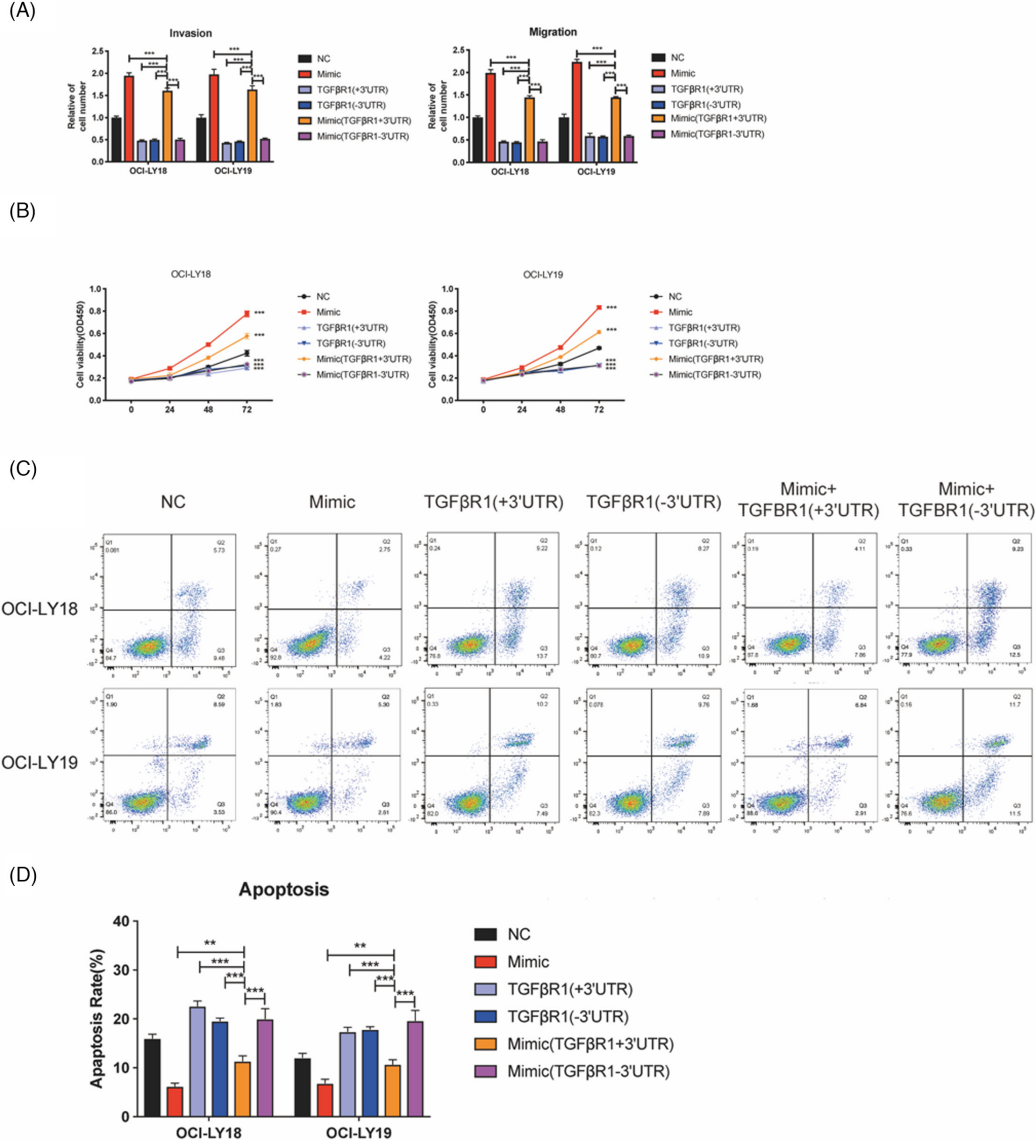# Corrigendum

**DOI:** 10.1002/jcla.24640

**Published:** 2022-09-09

Tang et al. Pre‐miR‐27a rs895819 polymorphism and risk of diffuse large B‐cell lymphoma. *J Clin Lab Anal*. 2020;34(3): e23088. doi: 10.1002/jcla.23088

We note that Figure 4C appeared incorrectly, as errors were introduced during preparation of these figures. We declare that these corrections do not change the results or conclusions of this article.

The far‐right upper panels of Figure 4C present the apoptosis rate of OCI‐LY18 cell treated with miR‐27a mimic and expression vectors containing TGFBR1 3′UTR.However, the picture was repeated with Figure 2F (the right bottom panel), which probably due to all these pictures were extremely similar, and we made a mistake during the preparation of these figures. We have corrected the Figure 4C in the following figure.